# Potential Molecular Mechanism of the *NPPB* Gene in Postischemic Heart Failure with and without T2DM

**DOI:** 10.1155/2020/2159460

**Published:** 2020-08-03

**Authors:** Yao-Zong Guan, Rui-Xing Yin, Guo-Xiong Deng, Peng-Fei Zheng, Chun-Xiao Liu, Bi-Liu Wei

**Affiliations:** ^1^Department of Cardiology, Institute of Cardiovascular Diseases, The First Affiliated Hospital, Guangxi Medical University, Nanning, 530021 Guangxi, China; ^2^Guangxi Key Laboratory Base of Precision Medicine in Cardio-Cerebrovascular Disease Control and Prevention, Nanning, 530021 Guangxi, China; ^3^Guangxi Clinical Research Center for Cardio-Cerebrovascular Diseases, Nanning, 530021 Guangxi, China

## Abstract

**Background:**

This study is aimed at investigating natriuretic peptide B (*NPPB*) coexpression genes and their pathways involved in heart failure (HF) among patients both with and without type 2 diabetes mellitus (T2DM).

**Methods:**

The microarray dataset GSE26887, containing 19 postischemic HF patients' peripheral blood samples (7 with T2DM and 12 without T2DM), was examined to detect the genes coexpressed with *NPPB* using the corr.test function in the R packet. Furthermore, using online analytical tools, we determined the Kyoto Encyclopedia of Genes and Genomes (KEGG) pathway enrichment analysis, Gene Ontology (GO) annotation, and protein-protein interaction (PPI) network of the coexpression genes. The modules and hub genes of the PPI network were then identified using the Cytoscape software.

**Results:**

In patients with T2DM, a total of 41 biological processes (BP), 20 cellular components (CC), 13 molecular functions (MF), and 41 pathways were identified. Furthermore, a total of 61 BPs, 16 CCs, 13 MFs, and 22 pathways in patients without T2DM were identified. In both groups of patients, 17 BPs, 10 CCs, 6 MFs, and 13 pathways were enriched. We also identified 173 intersectional coexpression genes (63 positively, 106 negatively, and 4 differently coexpressed in patients with and without T2DM, respectively) in both types of patients, which were enriched in 16 BPs, 8 CCs, 3 MFs, and 8 KEGG pathways. Moreover, the PPI network (containing 237 edges and 170 nodes) with the top module significantly enriched in 4 BPs (tricarboxylic acid metabolic process, citrate metabolic process, tricarboxylic acid cycle, and aerobic respiration) and 3 pathways (citrate cycle, malaria parasite metabolic pathway, and AGE-RAGE signaling pathway in diabetic complications) was constructed. *DECR1*, *BGN*, *TIMP1*, *VCAN*, and *CTCF* are the top hub genes.

**Conclusions:**

Our findings may elucidate the functions and roles of the *NPPB* gene in patients with postischemic HF and facilitate HF management.

## 1. Introduction

Heart failure (HF) is a challenge for numerous cardiovascular specialists, as it affects both the health and quality of life of a tremendous number of patients. It is estimated that 26 million people worldwide suffer from HF, according to data from a prior survey [1]. Moreover, annual costs to treat and manage HF ranges from International Dollars (Int$) 2,496.00 to Int$ 84,434.00 per patient [2]. However, it is estimated that the in-hospital mortality ranges from 4% to 30% and that the all-cause 1-year mortality rates among patients with acute HF and patients with chronic HF were 23.6% and 6.4%, respectively [3]. The etiology of heart failure involves coronary artery disease, rheumatic heart disease, cardiomyopathies, hyperthyroidism, and so on. Among these diseases, ischemic heart failure is common, especially when caused by ST-segment elevation myocardial infarction [4]. Although the numbers of chest-pain centers and cardiac care units (CCUs) have been increasing and thus more patients have received timely and effective interventions, postischemic HF remains a challenge that cannot be neglected any further.

Type 2 diabetes mellitus (T2DM), which is an endocrine disease that mainly leads to vascular and nerve damage, is regarded as an equal-risk syndrome of coronary heart disease and accompanies patients for the rest of their lives [5]. It is reported that T2DM not only promotes the development of HF but also increases the risk of cardiovascular disease (CVD) 2- to 4-fold [6, 7]. It is estimated that more than 400 million persons are affected by T2DM worldwide, costing $1.3 trillion annually [8]. In addition, the coexistence of HF and T2DM is common, and in populations ranging from 33 years to 84 years, the prevalence of HF in people with T2DM was 12% [9]. Moreover, T2DM and HF mutually promote the development of each other, and it is more complicated to treat HF patients with T2DM [10].

Currently, the diagnosis of HF is mainly based on clinical manifestations. Fortunately, serum BNP (encoded by the *NPPB* gene) and NT-pro-BNP levels have greatly contributed to the proper diagnosis of HF [11]. BNP is mainly secreted by atrial myocytes and thus reflects the heart load. BNP can represent powerful biological effects, such as natriuresis, vasodilation, myocardial apoptosis inhibition, and modulation of immune and inflammatory responses of cardiac injury [12–14]. Some prior studies suggest that BNP can be used as a biomarker for prognosis in patients with HF, and it also participates in both occurrence and development of T2DM and ischemic cardiomyopathy [15, 16]. What's more, in diabetic patients, BNP can be used for screening the absence of left ventricular dysfunction [17]. Therefore, BNP is associated with HF as well as T2DM. However, results from early researches show us that serum BNP levels are higher in HF patients with diabetes than in HF patients without diabetes, while some others report the opposite result [18, 19]. It is not yet clear whether there are some shared and specific mechanisms of the *NPPB* gene in HF patients with and without T2DM.

In recent years, microarray sequencing technology has rapidly developed and has significantly assisted basic and clinical medicine. The Gene Expression Omnibus (GEO) database is a huge repository that stores a series of high-throughput microarray and next-generation sequence functional genomic datasets and is free for global researchers to use for mining purposes [20]. In this study, we aimed to further understand the function of the *NPPB* gene in HF patients and better inform HF management by detecting the *NPPB* coexpression genes and pathways enriched in patients with postischemic HF either with or without T2DM.

## 2. Methods

### 2.1. Affymetrix Microarray Data

**Figure 1:**
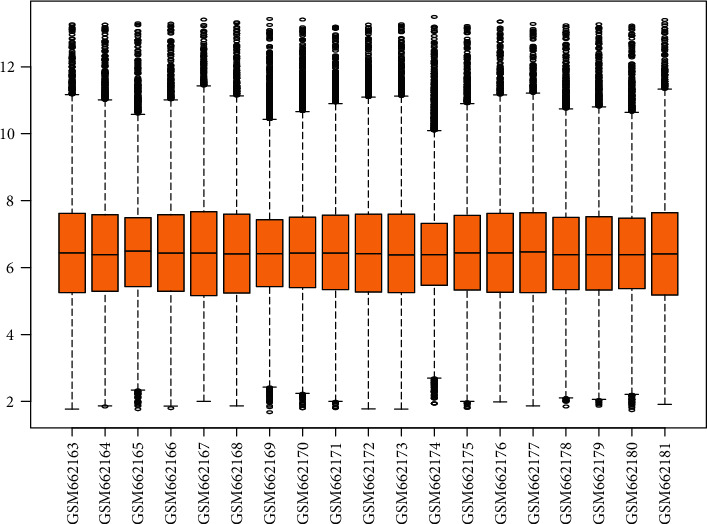
Normalization of gene expression. The orange box represents the expression of genes, and the black line in the box represents the median. The *x*-axis represents the sample name, and the *y*-axis represents the expression level.

The microarray dataset GSE26887 was retrieved from the GEO database. This dataset contained 24 samples from 19 patients with postischemic heart failure (7 with T2DM and 12 without T2DM) and 5 from control nonfailing hearts [21]. We recruited patients with postischemic heart failure either with T2DM (DHF group, *n* = 7) or without T2DM (nDHF group, *n* = 12) for analysis. The extracted data were normalized before further analysis in order to ensure the comparability of samples by the limma package that is available in the R platform [22] (Figure 1).

### 2.2. Identification of *NPPB* Coexpression Genes

A screening of coexpression genes for *NPPB* from the samples was performed by the corr.test function in R (version 3.6.1). Screening criteria were as follows: *P* < 0.05 and ∣Pearson correlation coefficient | ≥0.4. The online analytical tool Draw Venn Diagram (http://bioinformatics.psb.ugent.be/webtools/Venn/) was then used to determine the intersectional coexpression genes of both groups.

### 2.3. GO and KEGG Pathway Enrichment Analyses

The online database DAVID (version 6.8) [23] was used for GO and KEGG enrichment analyses [24, 25]. A *P* value of <0.05 was set as significant. The ggplot2 package was used for the visualization of the results in R (version 3.6.1).

### 2.4. Integration of the PPI Network

The STRING (version 10.5) database was used for evaluating the interactions among the coexpression genes, and a combined interaction score of >0.4 was set as significant [26]. In addition, the top 10 hub genes were identified using Cytoscape plugin cytoHubba (version 0.1) with the degree ratio ranking method. Furthermore, the MCODE and ClueGO apps in Cytoscape were used to identify the modules, namely the GO annotation and KEGG pathway enrichment analyses, respectively, of the PPI network [27].

## 3. Results

### 3.1. Identification of *NPPB* Coexpression Genes

**Figure 2:**
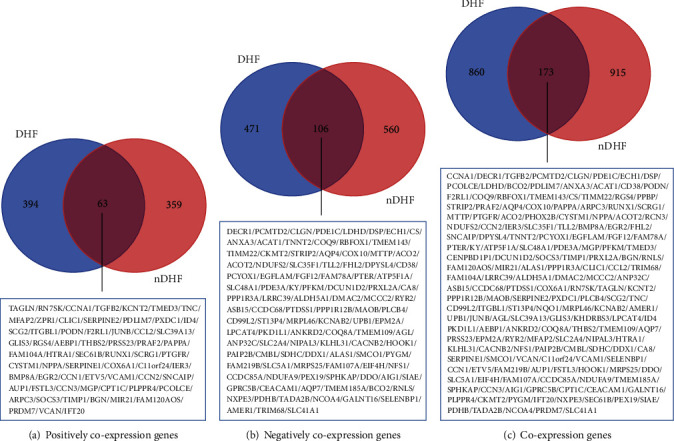
Venn diagram of coexpression genes. (a) Venn diagram of genes positively coexpressed with *NPPB*. (b) Venn diagram of genes negatively coexpressed with *NPPB*. (c) Venn diagram of *NPPB* coexpression genes. DHF: postischemic patients with T2DM. nDHF: postischemic patients without T2DM.

A total of 577 negatively coexpressed genes and 457 positively coexpressed genes in the DHF group were identified, along with 666 negatively coexpressed genes and 422 positively coexpressed genes in the nDHF group.  Figure 2 portrays 106 negatively and 63 positively coexpressed genes in both patient types, whereby 173 intersectional coexpression genes were screened out. Interestingly, of these intersectional coexpression genes, we found 3 genes (*CENPBD1P1*, *KHDRBS3*, and *PHOX2B*) that were positively coexpressed with *NPPB* in patients with T2DM, but negatively coexpressed in patients without T2DM, and 1 gene (*NQO1*) that was negatively coexpressed with *NPPB* in patients with T2DM, but positively coexpressed in patients without T2DM.

### 3.2. Functional GO and KEGG Pathway Enrichment Analyses

**Figure 3:**
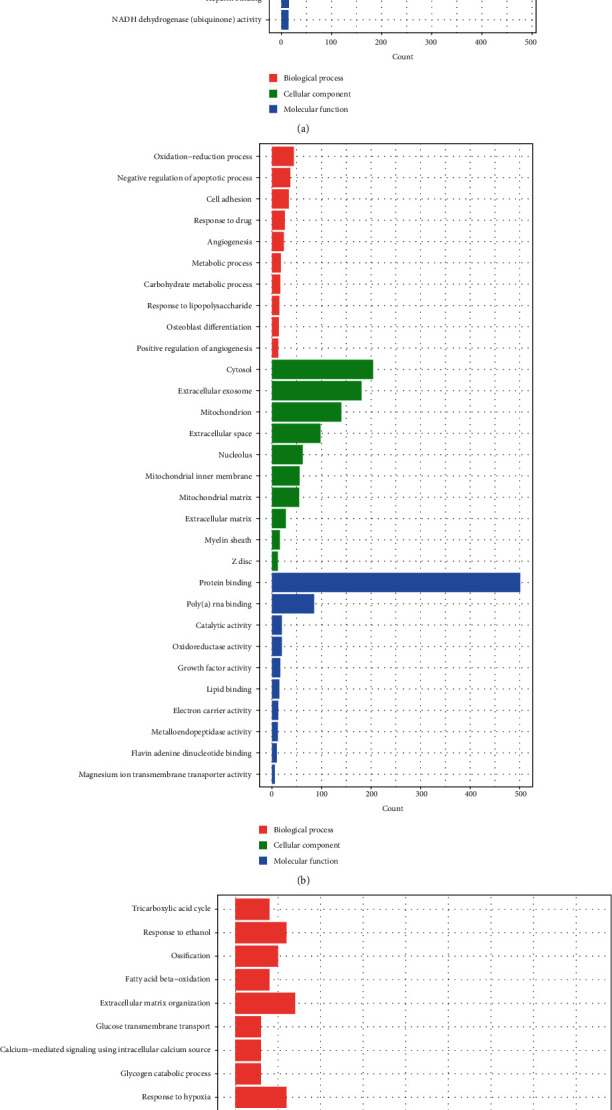
GO annotation of *NPPB* coexpression genes in postischemic heart failure patients: (a) in postischemic patients with T2DM and (b) in postischemic patients without T2DM. (c) Based on the intersectional coexpression genes of the two types of patients.

**Figure 4:**
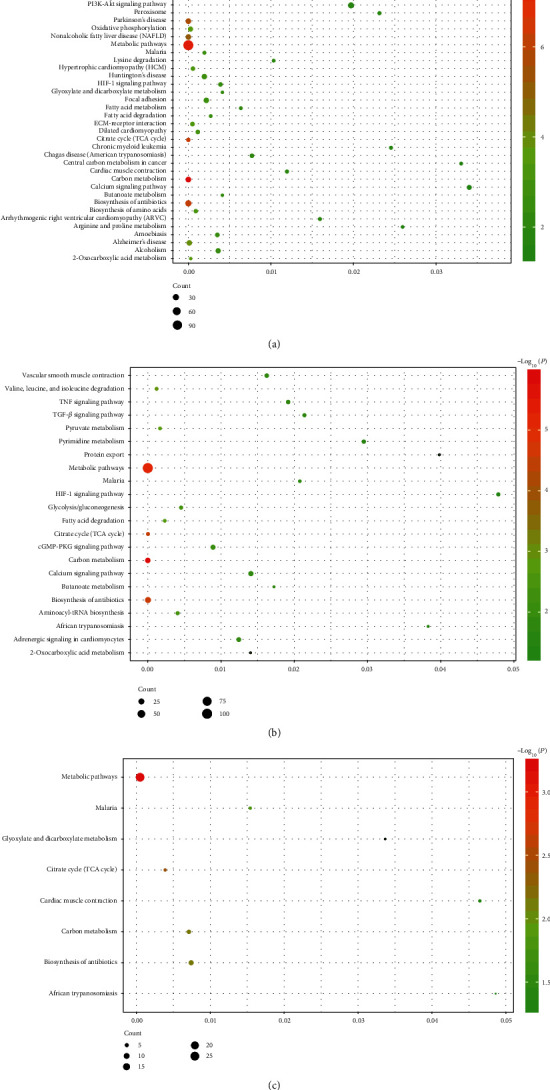
KEGG pathways of *NPPB* coexpression genes in postischemic heart failure patients: (a) in postischemic patients with T2DM and (b) in postischemic patients without T2DM. (c) Based on the intersectional coexpression genes of the two types of patients.

GO analyses revealed 41 BPs, 20 CCs, and 13 MFs in the DHF group, and 61 BPs, 16 CCs, and 13 MFs in the nDHF group (details in Tables S1 and S2). Due to the excessive number of enrichment analyses, the top seven BPs, CCs, and MFs were selected for visualization with *P* < 0.05 (Figures 3(a) and 3(b)). Furthermore, there were 10 BPs (fatty acid beta-oxidation, oxidation-reduction process, metabolic process, mitochondrial respiratory chain complex I assembly, glyoxylate metabolic process, ubiquinone biosynthetic process, positive regulation of cell growth, tricarboxylic acid cycle, cell adhesion, and aerobic respiration), 8 CCs (mitochondrial inner membrane, extracellular space, mitochondrion, extracellular matrix, myelin sheath, extracellular exosome, Z disc, and mitochondrial matrix), and 3 MFs (growth factor activity, protein binding, and electron carrier activity) enriched in both patient groups. There were 41 identified pathways in patients with T2DM (Figure 4(a)) and 22 in patients without T2DM (Figure 4(b)) (details in Tables S1 and S2). Moreover, common pathways are shown in Table 1.

The analyses further identified 16 BPs, 8 CCs, and 3 MFs that were enriched by intersectional coexpression genes in both patient groups (Figure 3(c)), and these genes mainly clustered in the following 8 pathways: the citrate cycle (TCA cycle), carbon metabolism, biosynthesis of antibiotics, malaria, glyoxylate metabolism, dicarboxylate metabolism, cardiac muscle contraction, and African trypanosomiasis (Figure 4(c)) (details in Table S3).

### 3.3. PPI Network Construction and Hub Gene Identification

**Figure 5:**
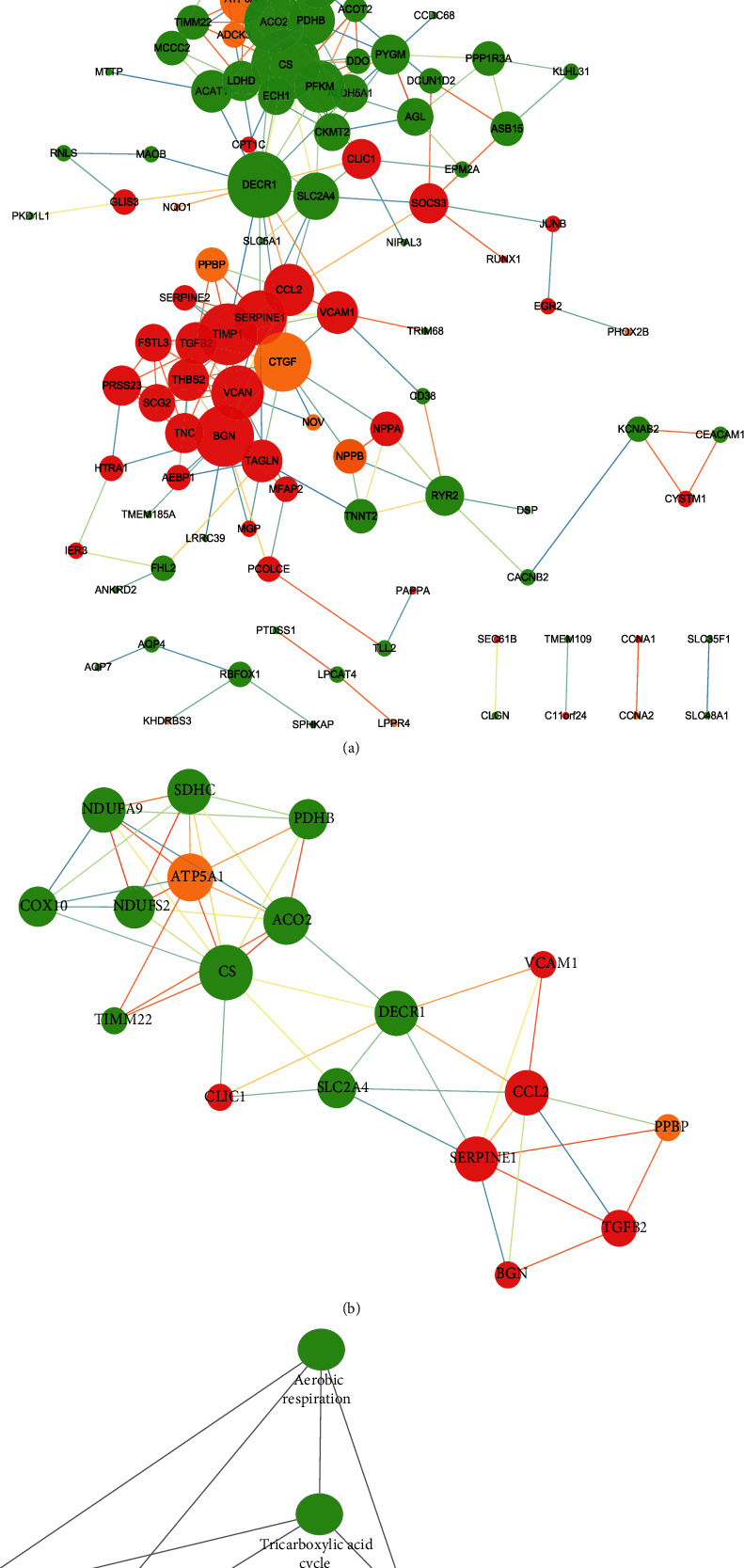
Protein-protein interaction (PPI) network of intersectional *NPPB* coexpression genes. (a) PPI network based on the intersectional *NPPB* coexpression genes of two types of patients. The red ball represents positive coexpression, while the green ball represents negative coexpression. The thickness of the line represents the strength of the correlation. (b) Module identified with a cutoff criterion of MCODE score > 5. (c) Biological process and KEGG pathways enriched in the module. (d) Top 10 hub genes. The color depth represents the ranking of hub genes. The sequence of colors is red-orange-yellow from high ranking to low ranking.

**Figure 6:**
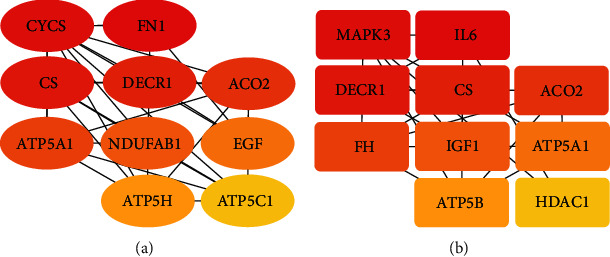
Top 10 hub genes of the PPI network. (a) Top 10 hub genes of the PPI network based on the *NPPB* coexpression genes in the DHF group. (b) Top 10 hub genes of the PPI network based on the NPPB coexpression genes in the nDHF group. The sequence of colors is red-orange-yellow from high ranking degree to low ranking degree.

As Figure 5 shows, the interactions among intersectional coexpression genes were displayed by a PPI network with 273 edges and 170 nodes. This finding was saved in *TSV* format and then imported into Cytoscape for visualization. With a cutoff criterion of a degree that is >5 and a K − core > 5, only one module with 4 BPs (tricarboxylic acid metabolic process, citrate metabolic process, tricarboxylic acid cycle, and aerobic respiration) and 3 pathways (citrate cycle, malaria parasite metabolic pathway, and AGE-RAGE signaling pathway in diabetic complications) significantly enriched was identified. With the degree ratio ranking method, the top 10 hub genes of this PPI network were also identified (*CS*, *DECR1*, *ACO2*, *BGN*, *TIMP1*, *CTGF*, *VCAN*, *SERPINE1*, *SDHC*, and *CCL2*). With the same cutoff criterion, a PPI network that consists of 953 nodes and 4,946 edges of *NPPB* coexpression genes in the DHF group, and a PPI network of 1,009 nodes and 4,245 edges in the nDHF group were also constructed. The top 10 hub genes of the former were *CYCS*, *FN1*, *CS*, *DECR1*, *ACO2*, *ATP5A1*, *NDUFAB1*, *EGF*, *ATP5H*, and *ATP5C1*, while the top 10 hub genes of the latter were *CS*, *DECR1*, *BGN*, *TIMP1*, *ACO2*, *CTGF*, *VCAN*, *SERPINE1*, *CCL2*, and *SDHC* (Figures 6(a) and 6(b), respectively). The visualization of these two PPI networks and their modules were concluded in Supplementary Materials (Figures S1–S4).

### 3.4. Verification of Hub Genes

Another dataset, GSE5406, containing 210 left ventricular myocardium samples (86 with idiopathic dilated cardiomyopathy, 108 with ischemic cardiomyopathy, and 18 unused donor hearts) was downloaded from the GEO database to verify the hub genes. We selected the heart failure with advanced ischemic cardiomyopathy samples (*n* = 108) for *NPPB* coexpression gene analysis with the same method as described in Methods. The correlation values of the *NPPB* coexpression gene in the GSE5406 dataset and in the GSE26887 dataset are shown in Table 2. Except for *CCL2*, other hub genes are coexpressed to *NPPB* with *P* < 0.05 and ∣Pearson correlation coefficient | >0.2. Both the positive coexpressed relationship and negative coexpressed relationship correspond.

## 4. Discussion

Although living and medical standards have undergone remarkable progress, heart failure remains a worldwide challenge, which costs countries a tremendous amount of money and affects the quality of life of patients at different degrees. Ischemic cardiomyopathy is one of the most common causes of heart failure; moreover, a portion of these patients also suffer from other diseases, such as type 2 diabetes mellitus, which complicates the treatment interventions for heart failure. Angiotensin-converting enzyme inhibitors, beta-blockers, diuretics, positive inotropic drugs, and cardiac resynchronization therapy (CRT) have been widely used in postischemic heart failure therapy, but quite a few patients inevitably go into end-stage heart failure for a variety of reasons [8]. Thus, they experience repeated hospitalizations, a severe decline in quality of life, complications in other organs, and even death. Serum BNP, encoded by *NPPB*, is secreted primarily by atria muscle cells, and BNP level increases when the heart is overloaded. It has been applied in clinics as a diagnostic and prognostic biomarker of HF for a long time, which is a great achievement [28]. Besides, BNP is also reportedly associated with the development of T2DM, and in turn, diabetes affected its expression in patients with HF. Some early researches reveal that the serum BNP level in HF patients without diabetes is higher than that in HF patients with diabetes, while other researches report the opposite result. Up to now, the mechanism is still completely clear. In this study, *NPPB* coexpression genes and their GO and KEGG pathways were identified in postischemic HF with T2DM and without T2DM, respectively, in order to further understand the potential mechanism of *NPPB* in postischemic HF patients with and without T2DM.

Heart failure is the result of the contradiction between the supply and demand of oxygen, blood, and energy, and the tricarboxylic acid cycle (TCA cycle) and mitochondrial respiratory transport chain are important links in glycolysis. As screened by the Venn diagram, a total of 63 positively coexpressed genes were identified. Carnitine palmitoyl transferase 1 (*CPT1*) encodes an important enzyme in the body, involved in fatty acid metabolism. As a subtype of *CPT1*, *CPT1C* can promote cell survival under metabolic stress conditions [29]. Furthermore, HtrA serine peptidase 1 (*HTRA1*) encodes a protein that is suggested to be a cell growth regulator, and its loss impairs smooth muscle cell maturation [30]. In a previous research, hypermethylation of the *SOCS3* gene could be an underlying mechanism of intimal hyperplasia and restenosis. *SOCS3* can also regulate cavin-1 function by enhancing its stability and consequently maintaining expression levels of caveolin-1 and cell surface caveolae. Moreover, proteins encoded by cavin-1 are also believed to modify lipid metabolism and insulin-regulated gene expression [31, 32]. In terms of vascular function, *CCN1* not only functions as an inhibitory regulator of SMC muscle contractility through inhibiting actomyosin interactions but also regulates TNF-*α* induced vascular endothelial cell apoptosis [33]. The *PDLIM7* gene product is involved in actin filament-associated complex assembly, which is essential for the transmission of ret/ptc2 mitogenic signaling. In addition, its expression is positively correlated to typical smooth muscle cell markers in atherosclerosis plaques, and PDLIM7 silencing in vitro led to downregulation of smooth muscle cell (SMC) markers, disruption of actin cytoskeleton, decreased cell spreading, and increased proliferation [34]. The data from Thomsen et al. suggested that in patients with ischemic heart disease, increased plasma MGP levels are indicative of a progressing calcification process [35]. Moreover, protease-activated receptor 2 (*PAR2*) in microvascular endothelial cells is indispensable for vascular stability, and its deficiency attenuates atherosclerosis [36, 37]. The abovementioned genes mainly play a role in energy supply and metabolism, cell proliferation and apoptosis, and vessel function and development, and they have been reportedly associated with blood and oxygen supply and cardiac remodeling in patients with HF.

On the other hand, a Venn diagram allowed identifying 106 genes negatively coexpressed with *NPPB*. Coq8p and human COQ8A are related to CoQ biosynthesis, and acute inhibition of Coq8p is sufficient to cause CoQ deficiency and respiratory dysfunction [38]. *NDUFS2* and *NDUFA9* encode compound I subunits in the mitochondrial membrane respiratory chain, while *SDHC* encodes compound II subunits. Also, *DECR1* encodes an enzyme, referred to as NADPH, which provides H^+^ ions for NAD^+^ and then converts to NADH to participate in the respiratory chain. In addition to the respiratory chain, the TCA cycle also features several genes that are mainly active in its processes [39]. *PDHB* encodes a pyruvate dehydrogenase compound, which catalyzes the conversion of pyruvate into acetyl-CoA and carbon dioxide for the TCA cycle. Citrate synthase, which is encoded by *CS*, catalyzes citric acid synthesis from oxaloacetic acid and acetyl-CoA; furthermore, citric acid synthesis by oxaloacetic acid and acetyl-CoA is catalyzed by cisaconitum, which is encoded by *ACO2*. *ALAS1* encodes mitochondrial enzymes that catalyze rate-limiting steps in the heme (iron protoporphyrin) biosynthesis pathway. In the context of cell proliferation and vascular function, Yan reported that in senescent vascular SMCs, *PDE1A* and *PDE1C* mRNA levels are significantly upregulated, and cellular senescent makers were reduced when *PDE1* was inhibited [40]. Data from Begum et al. suggest that therapies specifically aimed at inhibiting the PDE3A isoform may lead to the amelioration of excessive vascular SMC growth and decrease the atherosclerosis process [41]. Thus, the abovementioned genes are mainly involved in the regulation of the tricarboxylic acid cycle and respiratory transport chain in terms of energy supply and maintain the normal function of vascular SMC. Finally, *CACNB2*, *KCNAB2*, and *TIMM22* encode subunits that participate in dysfunctional voltage-gated channels that may be associated with arrhythmia events rather than aggravated heart failure [42, 43]. Thus, these are factors that are associated with the development of heart failure.

In addition, Table 1 shows us the shared pathway that occurs in both postischemic HF with or without T2DM. Most of the pathways are related to metabolism, such as the following: the citrate cycle (TCA cycle); butanoate, carbon, pyruvate, and 2-oxocarboxylic acid metabolism; and valine, leucine, isoleucine, and fatty acid degradation. Figure 5 shows that it is similar to the pathways of the intersectional coexpression genes and the genes of the module that are enriched within the PPI network. Furthermore, the HIF-1 signaling pathway is a hot topic that researchers focus on. In M1 macrophages, HIF-1*α* activates the expression of the iNOS gene, increasing nitric oxide synthesis, which expands the blood vessels. As such, in hypoxia macrophages, the HIF-1*α*-pyruvate dehydrogenase kinase (PDK1) axis can induce active glycolysis [44]. In addition, an investigation from Chen et al. [45] suggests that HIF-1*α* and FoxO3a show synergistic effects of cardiomyocyte apoptosis under hypoxia, as well as elevated glucose levels. Another pathway, the TGF-*β* signaling pathway, is also a popular hot topic. TGF-*β* is a multifunctional cytokine, which can regulate the macrophage phenotype, promote T_reg_ cell activation, and reduce adhesion molecule synthesis by endothelial cells that lend a powerful anti-inflammatory effect [46]. Data from the study by Kim et al. show us that the TGF-*β* signaling pathway plays an important role in the regulation of cardiac fibrosis [47]. Lastly, as a classical pathway, the calcium signaling pathway was also found in both the DHF and nDHF patient groups. Ca^2+^ participates in excitation-contraction coupling, regulating myocardial contraction and diastole. In addition, it also takes part in the regulation of the cardiomyocyte action potential, which plays an essential role in managing heart rhythm [48, 49]. Thus, regulation disorders of the calcium signaling pathway will lead to heart rate disorders, myocardial contraction, and adrenal dysfunction. The abovementioned pathways affect patients with postischemic heart failure in terms of energy supply, metabolism, inflammation, and myocardial fibrosis.

Compared to HF patients without T2DM, the *NPPB* coexpression genes were enriched in several other pathways, such as arrhythmogenic right ventricular cardiomyopathy (ARVC), dilated cardiomyopathy, hypertrophic cardiomyopathy (HCM), cardiac muscle contraction, alcoholism, and the PI3K-Akt signaling pathway. The former three are different types of cardiomyopathy, and they mainly affect the morphology and function of ventricular muscle cells, resulting in the deterioration of cardiac function [50]. Alcohol abuse may double the risk of chronic HF compared to those who never had alcohol abuse [51], and the BNP level may increase markedly [34]. In context to the PI3K-Akt signaling pathway, it has been revealed to be involved in the expression level of BNP and in the cardioprotection afforded by BNP infusion [52, 53]. Thus, these pathways and the genes they enriched would affect the level of BNP and the development of HF.

Although we use the microarray dataset to help us identify the *NPPB* coexpression genes and pathways they enriched in postischemic HF patients, either in patients with T2DM or without T2DM, the occurrence and development of HF is complex, and a variety of aspects should be taken in consideration in the management of HF. We hope our findings could give a hand to a deeper understanding of the role and function of the *NPPB* gene in HF patients and provide aspects for the research and management of HF in the future.

## 5. Conclusions

The *NPPB* coexpression genes were used to identify the potential molecular mechanisms of the *NPPB* gene in DHF and nDHF patients in this study. Our findings may help elucidate the roles of *NPPB* and its coexpression genes in postischemic heart failure and serve as a clinical reference for future HF management. However, further research is required to validate the role of these coexpression genes and pathways.

## Figures and Tables

**Table 1:** The shared pathways enriched by the *NPPB* coexpression genes in patients from the DHF group and the nDHF group.

Pathway	DHF			nDHF		
	Count (%)	*P* value	Gene	Count (%)	*P* value	Gene
Biosynthesis of antibiotics	33 (2.30)	6.34*E*‐07	BCAT1/LDHB/EHHADH/ALDOC/OGDHL/PGAM2/ECHS1/OGDH/ACAT1/PDHB/CMBL/GOT2/GOT1/IDH3G/ATIC/RGN/PDHA1/NSDHL/DLST/ACO2/CS/IDH3B/PFKM/PYCR1/G6PD/HMGCS2/SDHC/DLD/PCYOX1/PCCB/MDH2/PRPS2/ACAA1	29 (1.86)	2.69*E*‐05	SC5D/ADH5/ACSS2/PSPH/ACAT1/HADHA/PDHB/CMBL/ACSS1/IDH2/SUCLA2/HADH/FH/ACAA2/ACO2/CS/BCKDHB/FBP1/DLAT/PFKM/AK9/IDH3A/SDHA/GPI/ALDH7A1/SDHC/PGM1/ALDH2/PCYOX1
Metabolic pathways	116 (8.07)	3.08*E*‐08	LDHB/EHHADH/NDUFAB1/PGAM2/OGDH/PDHB/HIBADH/MTHFD1L/CMBL/GOT2/GOT1/IDH3G/ST3GAL5/ST3GAL4/RGN/PDHA1/BST1/LPCAT4/CD38/PYCR1/NNT/DLD/PCCB/PRPS2/MDH2/ACAA1/ACADSB/ME3/ALDOC/ACSBG2/ACAT1/B4GALT6/UPB1/MAOB/SPHK1/ACMSD/IDH3B/ACACB/NDUFV3/GGT5/HMGCS2/PLCG2/COX6A1/BCO1/PHYKPL/UQCRC2/ETNPPL/COX11/COX10/OGDHL/ACOT2/QARS/MTHFD1/MCCC2/ALAS1/NDUFS4/PLCB4/XYLT1/MCEE/MCCC1/PIGC/AGPAT4/PTDSS1/NDUFS3/NDUFS2/NDUFS1/HYAL1/ACO2/ALDH5A1/NADSYN1/COX4I1/PIGS/PFKM/NDUFA10/PIGO/COQ6/ACADVL/COQ2/G6PD/CKM/PRDX6/PANK1/INPP4A/BCAT1/NDUFB6/ALDH18A1/NDUFB8/NDUFB9/NFS1/ECHS1/COMT/AZIN2/TYMS/ATIC/DHCR7/ALDH1A3/CKMT2/CYP26B1/GALNT16/BDH1/AGL/INPP5A/NSDHL/DLST/NDUFA2/NDUFA8/NDUFA9/NDUFA6/CS/MPI/PYGM/SDHC/AKR1B1/LIPG/DPM2/SCP2	105 (6.75)	7.14*E*‐06	SC5D/GNPDA1/PTGS2/CNDP2/DTYMK/ACSS2/PDHB/CMBL/FAHD1/ACSS1/CPOX/HADH/GPT2/ACAA2/FBP1/QDPR/LPCAT4/EARS2/CD38/CHPF/PGM1/MGAT5/GATB/GATC/LALBA/COASY/GCNT2/GLUD2/ACAT1/HADHA/POLE2/IDH2/DHODH/CDA/AMD1/FH/MGAT4B/MOCS2/B4GAT1/UPB1/MAOB/DLAT/RIMKLA/IDH3A/AK9/ALDH2/COX6A1/NNMT/COX10/ACOT2/PIP5K1B/PSPH/MCCC2/ST6GALNAC6/ALAS1/PLCB4/ACAD8/PTDSS1/SUCLA2/AGPAT3/NDUFS2/POLR1E/CYP11A1/ACO2/ALDH5A1/ACADS/ATP6V1H/PFKM/COQ7/ST6GALNAC1/MAN2A2/ALDH7A1/MTMR14/ADK/GPAM/UQCRB/POLR2H/TYRP1/NFS1/ADH5/ATP6V1G1/PLPP1/NDUFB1/CBR1/CKMT2/PLCD3/GALNT16/UCK2/PAFAH1B2/PDHX/AGL/EBP/POLR3F/MTHFD2L/B3GALT2/NDUFA9/BCKDHB/CS/POLR3GL/DGKI/SDHA/GPI/PYGM/SDHC/LTA4H
Citrate cycle (TCA cycle)	12 (0.83)	5.08*E*‐07	DLST/IDH3G/ACO2/SDHC/DLD/OGDHL/CS/IDH3B/PDHA1/OGDH/MDH2/PDHB	10 (0.64)	3.01*E*‐05	SDHA/ACO2/SDHC/CS/IDH2/DLAT/SUCLA2/IDH3A/PDHB/FH
Malaria	10 (0.70)	1.14*E*‐03	VCAM1/CCL2/COMP/TGFB3/THBS1/THBS2/IL10/TGFB1/SDC2/TGFB2	8 (0.51)	2.08*E*‐02	VCAM1/ITGAL/IL6/CCL2/IL18/THBS2/SELE/TGFB2
Valine, leucine, and isoleucine degradation	14 (0.97)	1.89*E*‐06	BCAT1/ACADSB/EHHADH/ECHS1/ACAT1/HIBADH/MCCC2/HMGCS2/MCCC1/MCEE/DLD/AACS/PCCB/ACAA1	10 (0.64)	1.20*E*‐03	ACAA2/MCCC2/ALDH7A1/ACADS/BCKDHB/ALDH2/ACAD8/HADH/ACAT1/HADHA
Butanoate metabolism	7 (0.49)	4.12*E*‐03	HMGCS2/ALDH5A1/EHHADH/ECHS1/AACS/BDH1/ACAT1	6 (0.39)	1.72*E*‐02	L2HGDH/ALDH5A1/ACADS/HADH/ACAT1/HADHA
Carbon metabolism	26 (1.81)	5.38*E*‐09	ME3/EHHADH/ALDOC/OGDHL/ECHS1/PGAM2/OGDH/ACAT1/PDHB/GOT2/IDH3G/GOT1/MCEE/RGN/PDHA1/DLST/ACO2/CS/IDH3B/PFKM/G6PD/SDHC/DLD/PCCB/MDH2/PRPS2	22 (1.41)	1.34*E*‐06	ACO2/ACADS/GLUD2/CS/ADH5/FBP1/PFKM/DLAT/PSPH/ACSS2/ACAT1/HADHA/IDH3A/PDHB/SDHA/GPI/ACSS1/SDHC/IDH2/SUCLA2/GPT2/FH
HIF-1 signaling pathway	14 (0.97)	3.89*E*‐03	CAMK2G/RPS6/PDHB/TIMP1/RBX1/CDKN1A/PLCG2/SERPINE1/TEK/PDHA1/PIK3R3/EGF/NPPA/AKT2	11 (0.71)	4.79*E*‐02	PDK1/IL6/BCL2/MAPK3/EDN1/SERPINE1/IGF1/IFNGR2/NPPA/PDHB/TIMP1
Pyruvate metabolism	9 (0.63)	1.96*E*‐03	LDHB/ME3/DLD/LDHD/PDHA1/ACACB/ACAT1/MDH2/PDHB	9 (0.58)	5.79*E*‐03	ALDH7A1/ACSS1/LDHD/ALDH2/DLAT/ACSS2/ACAT1/PDHB/FH
TGF-*β* signaling pathway	11 (0.77)	2.50*E*‐02	LTBP1/GDF6/TGFBR1/TGFB3/ID4/BMPR1B/THBS1/TGFB1/TGFB2/BMP8A/RBX1	11 (0.71)	2.14*E*‐02	INHBB/SMAD9/E2F5/SMAD7/SMAD6/MAPK3/ID4/ID3/TGFB2/BMP8A/BMP6
Calcium signaling pathway	18 (1.25)	3.40*E*‐02	ORAI2/ADORA2B/ERBB4/CAMK2G/SPHK1/HTR4/PTGFR/VDAC3/VDAC1/CD38/PLCB4/ATP2A2/P2RX1/PDE1C/P2RX3/PLCG2/RYR2/F2R	19 (1.22)	1.41*E*‐02	SLC25A4/SLC25A5/MYLK3/PHKA1/PTGFR/VDAC2/GRM1/CD38/AGTR1/ADRB2/PLCB4/ADRB1/PDE1C/AVPR1A/PLCD3/ADRA1A/RYR2/CHRNA7/HTR2B
Fatty acid degradation	9 (0.63)	2.71*E*‐03	CPT1C/ACADVL/ECI2/ACADSB/EHHADH/ACSBG2/ECHS1/ACAT1/ACAA1	9 (0.58)	2.32*E*‐03	CPT1C/ACAA2/ALDH7A1/ACADS/ADH5/ALDH2/HADH/ACAT1/HADHA
2-Oxocarboxylic acid metabolism	7 (0.49)	2.85*E*‐04	GOT2/BCAT1/GOT1/IDH3G/ACO2/CS/IDH3B	5 (0.32)	1.40*E*‐02	ACO2/CS/IDH2/GPT2/IDH3A

**Table 2:** Verification of the hub genes.

Hub gene	Cor	*P*	Cor	*P*	Cor	*P*
	GSE5406		GSE26887	DHF	GSE26887	nDHF
*DECR1*	-0.48	<0.01	-0.83	0.02	-0.74	<0.01
*BGN*	0.51	<0.01	0.87	0.01	0.59	0.04
*TIMP1*	0.44	<0.01	0.89	<0.01	0.76	<0.01
*VCAN*	0.50	<0.01	0.81	0.03	0.61	0.04
*CTCF*	0.50	<0.01	0.95	<0.01	0.79	<0.01
*CS*	-0.35	<0.01	-0.84	0.02	-0.60	0.04
*ACO2*	-0.34	<0.01	-0.86	0.01	-0.62	0.03
*SERPINE1*	0.38	<0.01	0.89	<0.01	0.68	0.01
*SDHC*	-0.21	0.03	-0.92	<0.01	-0.64	0.03
*CCL2*	0.08	0.39	0.85	0.02	0.69	0.01

Cor: Pearson correlation coefficient.

## Data Availability

The datasets used and/or analyzed during the current study are available from the Gene Expression Omnibus repository (https://www.ncbi.nlm.nih.gov/geo/query/acc.cgi?acc=GSE26887; https://www.ncbi.nlm.nih.gov/geo/query/acc.cgi?acc=GSE5406).

## Abbreviations

GEO: Gene Expression Omnibus
GO: Gene Ontology
KEGG: Kyoto Encyclopedia of Genes and Genomes
MCODE: Molecular complex detection
PPI: Protein-protein interaction
BP: Biological processes
CC: Cellular components
MF: Molecular functions
T2DM: Type 2 diabetes mellitus
HF: Heart failure
TCA: Tricarboxylic acid cycle
SMC: Smooth muscle cells.

## Supplementary Materials

Supplementary 1 Figure S1: Protein-protein interaction (PPI) network of NPPB coexpression genes in the DHF group. The red ball represents positive coexpression, while the green ball represents negative coexpression. The thickness of the line represents the strength of the correlation.

Supplementary 2 Figure S2: Modules identified from the PPI network of NPPB coexpression genes in the DHF group. The red ball represents positive coexpression, while the green ball represents negative coexpression. The thickness of the line represents the strength of the correlation. (A–F) Module 1-module 6 are identified with a cutoff criterion of MCODE score > 5.

Supplementary 3 Figure S3: PPI network of NPPB coexpression genes in the nDHF group. The red ball represents positive coexpression, while the green ball represents negative coexpression. The thickness of the line represents the strength of the correlation.

Supplementary 4 Figure S4: Modules identified from the PPI network of NPPB coexpression genes in the nDHF group. The red ball represents positive coexpression, while the green ball represents negative coexpression. The thickness of the line represents the strength of the correlation. (A–C) Module 1-module 3 are identified with a cutoff criterion of MCODE score > 5.

Supplementary 5 Table S1: the GO and KEGG pathways in DHF patients.

Supplementary 6 Table S2: the GO and KEGG pathway in nDHF patients.

Supplementary 7 Table S3: the GO and KEGG pathways in both groups.
