# Supplementary figures and images for: Potential Molecular Mechanism of the NPPB Gene in Postischemic Heart Failure with and without T2DM
Source: Biomed Res Int. 2020 Aug 3;2020:2159460. doi: 10.1155/2020/2159460 (PMC7424400; doi:10.1155/2020/2159460)

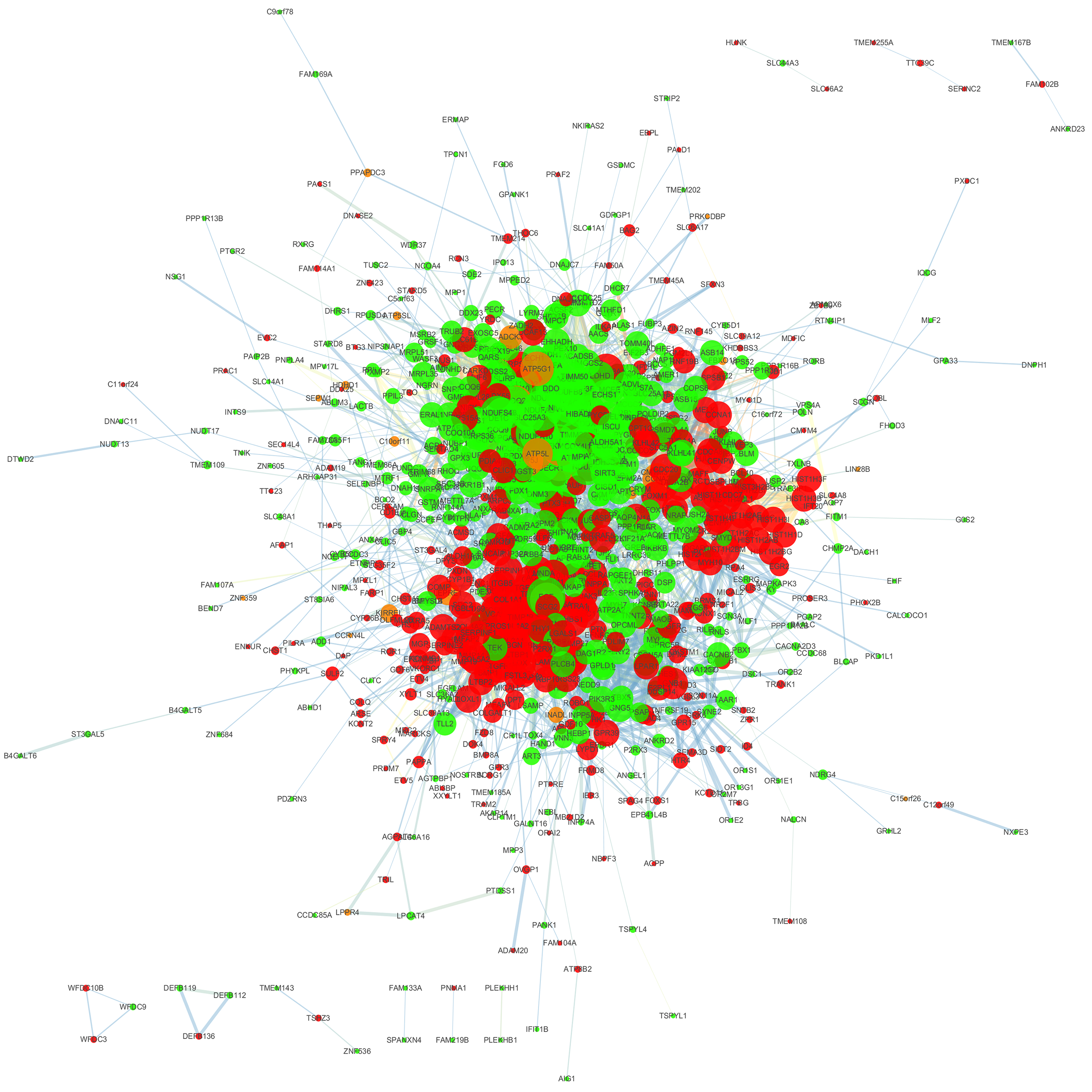

Supplement: Supplementary 1 — Figure S1: Protein-protein interaction (PPI) network of NPPB coexpression genes in the DHF group. The red ball represents positive coexpression, while the green ball represents negative coexpression. The thickness of the line represents the strength of the correlation. [file 2159460.f1.tif]

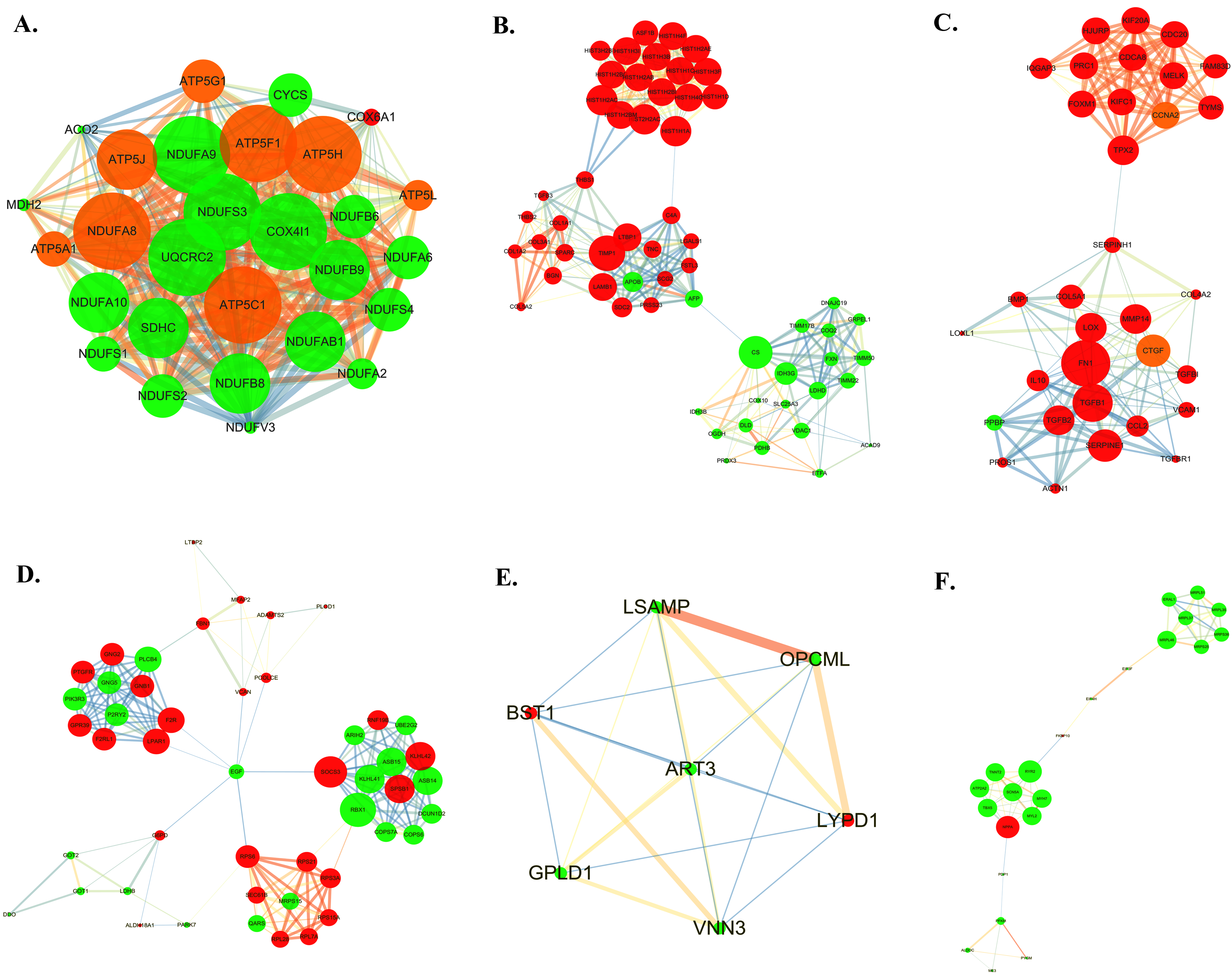

Supplement: Supplementary 2 — Figure S2: Modules identified from the PPI network of NPPB coexpression genes in the DHF group. The red ball represents positive coexpression, while the green ball represents negative coexpression. The thickness of the line represents the strength of the correlation. (A–F) Module 1-module 6 are identified with a cutoff criterion of MCODE score > 5. [file 2159460.f2.tif]

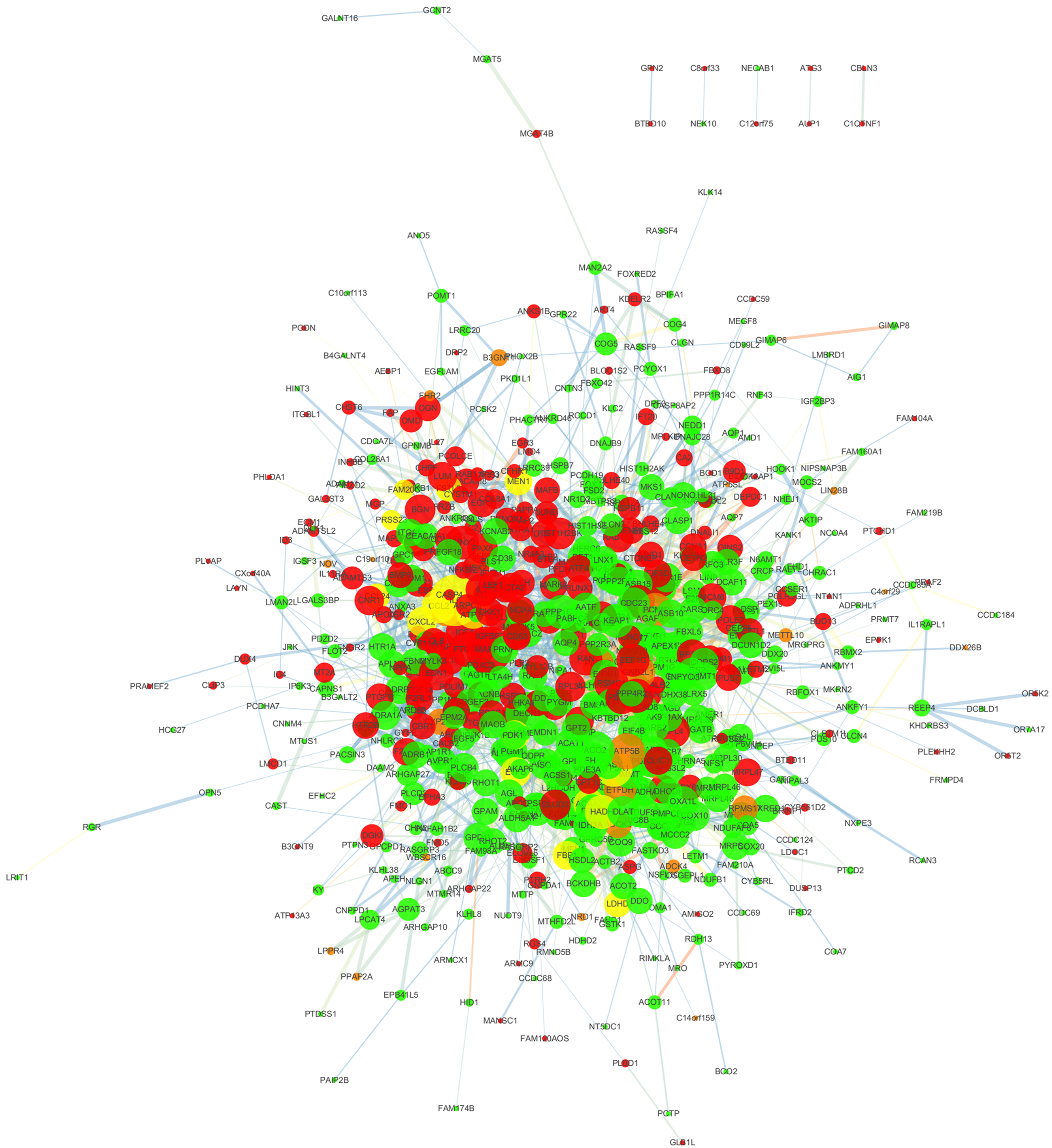

Supplement: Supplementary 3 — Figure S3: PPI network of NPPB coexpression genes in the nDHF group. The red ball represents positive coexpression, while the green ball represents negative coexpression. The thickness of the line represents the strength of the correlation. [file 2159460.f3.tif]

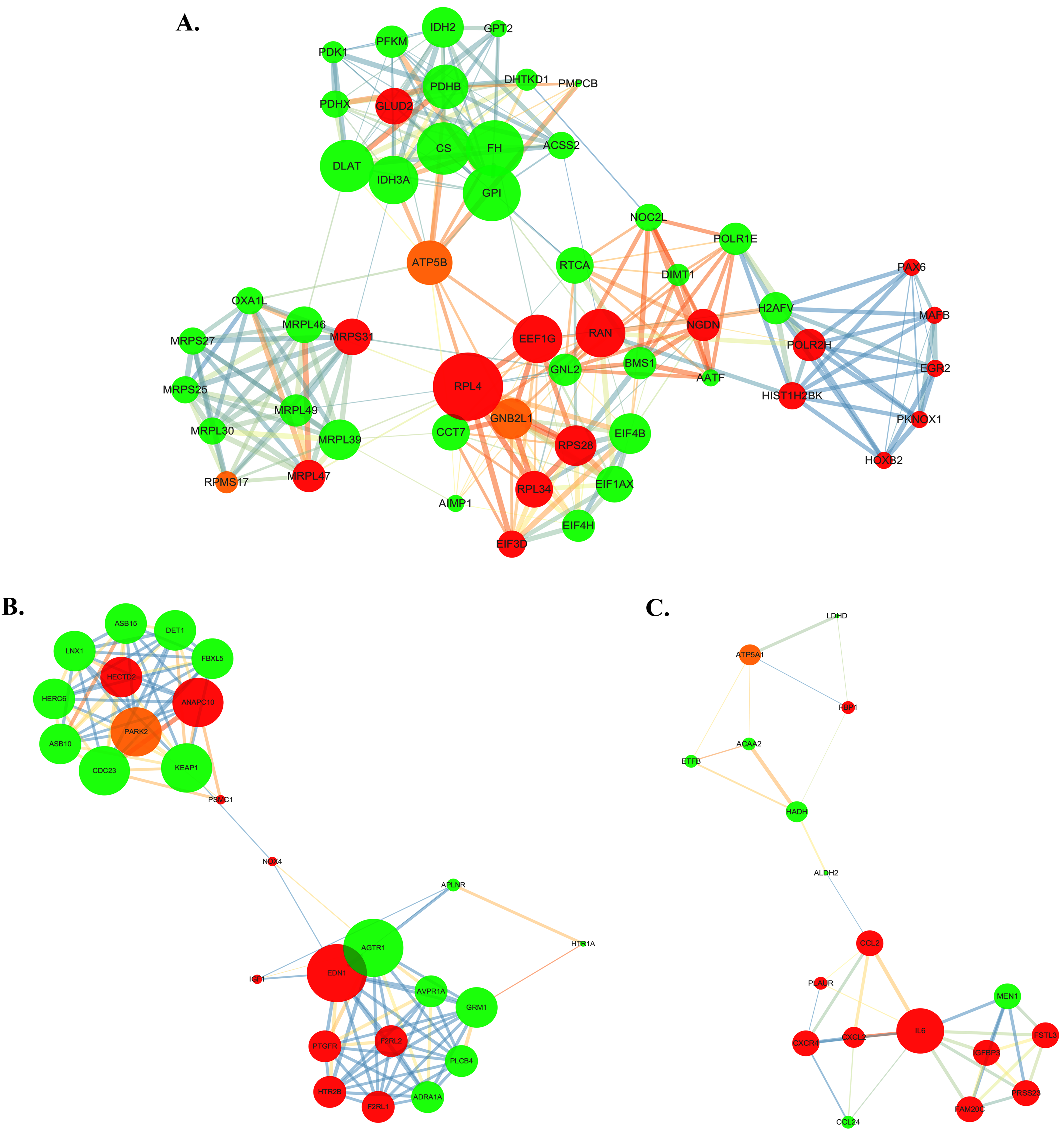

Supplement: Supplementary 4 — Figure S4: Modules identified from the PPI network of NPPB coexpression genes in the nDHF group. The red ball represents positive coexpression, while the green ball represents negative coexpression. The thickness of the line represents the strength of the correlation. (A–C) Module 1-module 3 are identified with a cutoff criterion of MCODE score > 5. [file 2159460.f4.tif]
